# How to evaluate intensive care unit performance during the COVID-19 pandemic

**DOI:** 10.5935/0103-507X.20200040

**Published:** 2020

**Authors:** Fernando Godinho Zampieri, Marcio Soares, Jorge Ibrain Figueira Salluh

**Affiliations:** 1 Programa de Pós-Graduação em Medicina Translacional e Departamento de Medicina Intensiva, Instituto D’Or de Pesquisa e Ensino - Rio de Janeiro (RJ), Brasil.; 2 Instituto de Pesquisa, HCor-Hospital do Coração - São Paulo (SP), Brasil.; 3 Programa de Pós-Graduação, Universidade Federal do Rio de Janeiro - Rio de Janeiro (RJ), Brasil.

One of the current definitions of an efficient intensive care unit (ICU) is being able to provide care that leads to a lower than expected mortality rate, at a lower use of resources (usually considering a lower use of resources as a surrogate for costs).^(1,2)^

The process of translating high-quality care into the largest number of survivors with the highest possible quality of life should be the goal of all ICUs.^(3)^Assessing ICU performance is, therefore, pivotal to identify outliers, targets for improvement, and bottlenecks in the process of care. Measuring the performance in ICUs is a cumbersome task, but despite its challenges, several methods have been successfully applied in the last three decades allowing performance evaluation and benchmarking.^(2,4)^ Intensive care unit performance is usually measured through assessment of the standardized mortality ratio (SMR - i.e., the ratio between the actual mortality and the average expected mortality, usually obtained by means of an illness severity score, such as the Simplified Acute and Physiology Score 3 (SAPS3) and the standardized resource use (SRU), usually measured as the ratio between the survivors length-of-stay (LOS) and the expected LOS, also estimated from a severity score. Therefore, two essential aspects of “performance”, namely success rate and resource use, are combined to provide an overall picture of the unit.^(4-6)^This, however, is engrained on several important limitations, including the limited performance of illness severity scores in some scenarios, only partially correctable by statistical adjustments.^(6)^

A recent and sudden changes in the *status quo* of ICUs^(7)^ were determined by the current COVID-19 pandemic, which represent a challenge to the ICU performance and its accurate measurement. In this “new normal scenario”, ensuring a good ICU performance is essential for several reasons: it is easier and cheaper to improve performance by increasing the bed turnover than creating an extra bed (involving all of its initial investments, staffing and materials, and medications related costs). Recent evidence show that improvement in the ICU efficiency and, therefore, its performance, can be attained by providing evidence-based care, including light sedation, low-tidal volume ventilation, and measures to reduce healthcare-associated infections.^(8)^ Adherence to evidence-based medicine (EBM) practices is the first obvious marker of a good performing ICU, and is a candidate for early performance assessment. Therefore, measuring and tracking adherence to EBM for measures and processes of care can provide insightful and actionable information.^(5,9)^

Of course, many pressing issues may hamper the attempts to measure and improve performance during the COVID-19 pandemic, including the abrupt shift in the ICU case-mix (e.g. increased severity and number of ventilated patients), need for changes in the whole ICU operation due to droplet precautions measures, costs increases due to additional personal protection equipment, and even a reduction of the available staff either due to illness or burnout. Finally, although data is starting to be published, we have no current tool to accurately predict either COVID-19 mortality or LOS. This represents a major limitation, not only for SMR/SRU but also this reduces the potential use of other metrics based on cumulative outcomes, such as variable-adjusted life displays. Much caution is needed if one aims at using SMR at this moment. Illness severity scores usually performed poorly when single conditions (including sepsis or acute respiratory distress syndrome) are considered.^(2,4,6)^ Additionally, larger periods (usually 2 or 3 months) are required to allow a relevant number of patients with hospital outcomes. Therefore, if SMR and SRU are to be used and benchmarked, they should not be considered alone, neither be solely based on their absolute values, as larger temporal trends will be required. We, therefore, advoke that other variables should be measured to better understand the outcomes and help ICU directors to identify where to invest and/or change practices, aiming to achieve better outcomes. A comprehensive, but pragmatic, understanding of the case-mix and resource use, and its benchmarking, can be both feasible and insightful, (Table 1) and focus on adherence to the process of care may add substantial value to an approach strictly focused on outcomes.

**Table 1:** What to measure when evaluating intensive care unit efficiency in the COVID-19 pandemic

Domain/measure	Advantages	Limitations	Usefulness to evaluate ICU performance during COVID-19 pandemic
**Outcomes and clinical characteristics**			
ICU and hospital mortality rates	Easy to measure, reproducible, clinically relevant	Very case-mix sensitive	Moderate: patients with COVID-19 may have long ICU stay and present with different degrees of severity, frequent mortality assessment may underestimate ICU performance
Length of stay	Easy to measure, a proxy of efficiency, reproducible	Affected by structure, may be lowered by transfers or early deaths	Low: should not be considered alone
Unplanned ICU readmissions	Easy to measure, reproducible, clinically relevant, an indirect marker of clinical process inside and outside ICU	Affected by structure (e.g.- step-down units), artificially lowered by transfers, and end of life care policies, uncertain effect on mortality. Can be affected by strain.	Low: may be affected by ICU occupation (readmission refusals); In ICUs strained with COVID-19 and wards unprepared to care for such complex patients this rate may increase
ICU acquired complications	Usual and valid indicators of quality of care; actionable as there are preventive measures that can be applied	Affected by case-mix, frequently under-reported, the applied definition may vary and result in a poor benchmarking application	High: if ICU complications are low it is conceivable that major processes of care are preserved
SMRs and SRUs	Usual indicators of performance, validate for ICUs in general	Need large patient sample and complete outcomes to be more reliable; usually loses performance in specific populations and has trends to increase when overall case-mix changes fast with sudden shifts in mortality	Low: data on large number of patients needs to accumulate before widespread use. Depends on a well-validated illness severity score
**Process of care**			
Adherence to the evidence-based process of care measures to reduce ICU-acquired complications	Traditional proxies EBM practices. May be important in COVID-19 due to its elevated risk of complications. Can reflect strain.	Uncertain effect on mortality can be tricky to measure at the bedside. May require specialized monitoring systems	Highest: can provide information on staff adherence and identify ICU overload in the context of worsening adherence to protocols
**Staffing patterns**			
Staffing patterns	Potentially associated with outcomes, easy to measure	Should be adjusted by risk and workload, extremely hard to measure and not fully amenable to interventions in a time of crisis	Uncertain: very dependent on local patterns, case-mix, and workload

ICU - intensive care unit; SMR - standardized mortality ratio; SRU - standardized resource use; EBM - evidence-based medicine.

COVID-19 pandemic represents an abrupt change in the ICU outcomes (“producing survivors” process), with a sudden shift in the input, changes in process care, lack of effective and specific treatment protocols, an exceptional speed in changes of ICU routines, among other factors. This situation can be aggravated by a lack of proper equipment to provide life support, especially in strained ICUs or resource-constrained scenarios. For some ICUs, the limiting factor can be lack of equipment, lack of staff, late patient referral, or all the above. An individual assessment of cases with unfavorable outcomes using simple Ishikawa (“fishbone”) diagrams may be useful, particularly early in the pandemic. However, as the cases accumulate, the evidence must come from larger series with proper analysis.

Additional ways to measure performance can be borrowed from economics, especially using the production-possibility frontier and data envelopment analysis.^(10)^ Data envelopment analysis is an interesting econometric process where inputs and outputs are considered, and a benchmark performed. This analysis is flexible in the sense it accommodates with different metrics; for example, inputs may include staff levels, available equipment for organ support, number of beds and number of requested admissions (and their respective average illness severity) and outputs can include the number of survivors, mechanical ventilation free-days, ICU-free days, etc. It can also aid the identification of potential restraining issues between units (Figure 1). This may be useful, even for ICU comparison of performance over time, and benchmarking with other units.

**Figure 1:**
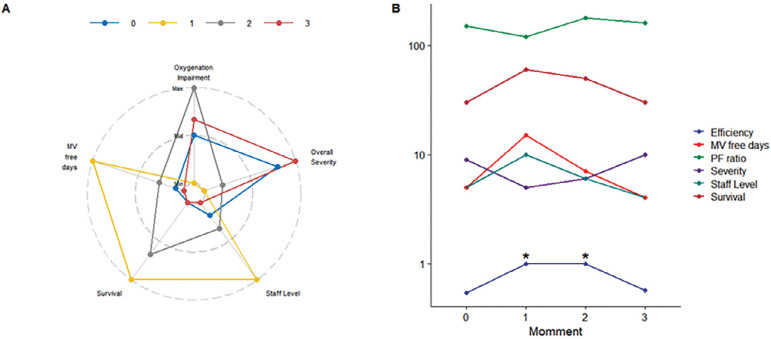
A novel model to measure intensive care unit performance. (A) A spider plot for a given intensive care unit at 4 different times (0 - 3) considering “inputs” (oxygenation impairment of admitted patients, average severity, staff level) and “outputs” (mechanical ventilation free days and survival). The same unit at 4 different points is shown. There are changes in illness severity, staff level, oxygenation over time, which results in differences in outputs. These trends together with relative efficiency are shown in panel (B). Note that at moments 1 and 2 the efficiency is maximized when compared with times 0 and 3 (marked with “*”), despite a reduction in staff level from 1 - 2 and fluctuations in severity. At point 3, performance seems to worsen (lower survival, less mechanical ventilation free days which are disproportional to increase in admission severity). Data envelopment could point that staff reduction is probably the limiting step in this toy example. Min - minimum; Max - maximum; MV - mechanical ventilation; PF - partial pressure arterial oxygen/fraction inspired oxygen.

## CONCLUSION

Measuring the ICU performance was never so important neither so difficult as during the COVID-19 pandemic. While few data on prognostic scores is available, therefore limiting the use of more traditional metrics, ICUs should focus on measuring indirect performance parameters, especially analyzing case-mix, outcomes, and the rate of adherence to best practices.
